# The Landscape of *Copia* and *Gypsy* Retrotransposon During Maize Domestication and Improvement

**DOI:** 10.3389/fpls.2019.01533

**Published:** 2019-12-18

**Authors:** Xiangbo Zhang, Yongwen Qi

**Affiliations:** Guangdong Bioengineering Institute (Guangzhou Sugarcane Industry Research Institute), Guangdong Key Lab of Sugarcane Improvement and Biorefinery, Guangzhou, China

**Keywords:** maize, retrotransposon elements, domestication and improvement, TE amplifed, TE contracted

## Abstract

The release of genomic sequences in the maize HapMap3 population provides an opportunity to study the genetic diversity of maize. In this study, retrotransposon insertion polymorphisms (RIPs) were mapped against the maize genome sequence. In total, 27 retrotransposon families were identified, and more than 170,000 RIPs were discovered in teosinte, landrace, and improved groups. Interestingly, the copy number of transposable elements (TEs) were more abundant in landrace groups than in teosinte or improved groups, suggesting that TEs experienced amplification during domestication and contraction during improvement. Landrace accessions exhibited higher TE insertion frequency compared to the other groups. Furthermore, the position of TE insertions were closer to genes and more abundant in the centromeres of landrace groups compared to the other groups. The three groups could be clearly distinguished by RIPs. These results demonstrate that TEs were amplified and contracted during maize domestication and improvement, respectively.

## Introduction

Transposable elements (TEs) are DNA sequences that can move from one position in the genome to another or generate repeats that are transferred to new positions. TEs occupy the majority of most eukaryotic genomes and are abundant in plants. In maize, TEs occupy ∼80% of the genome with genes embedded in a vast expanse of TEs (Schnable et al., 2015; Jiao et al., 2017; Springer et al., 2018; Sun et al., 2018). Among them, long terminal repeat (LTR) retrotransposons constitute the majority of TEs.

The process of domestication and improvement in crops has affected the TE content in crop genomes. In pepper, previous studies reported that the genetic diversity of sweet and large-fruited *Capsicum annuum* cultivars was narrowed during the domestication process (Aguilar-Melendez et al., 2009). By comparing the genomic sequences of cultivated pepper, Zunla-1, and its wild progenitor, Chiltepin, the pepper genome expanded ∼0.3 Mya and contained ∼81% repetitive sequences with a fast proliferation of retrotransposon elements (Qin et al., 2014). Clearly, TEs play an important role in pepper domestication and improvement. In maize, several genes were reported to be functionally associated with TE insertions (Qin et al., 2013; Mao et al., 2015; Huang et al., 2018). For example, a hopscotch element inserted at 60 kb upstream of teosinte branched1 (tb1), is associated with maize apical dominance (Anthony et al., 2011). Moreover, a CACTA-like TE inserted at 2 kb upstream of ZmCCT, results in maize photoperiod sensitivity (Qin et al., 2013).

With the advancement of next-generation sequencing technologies, researchers have uncovered new TE insertions in non-reference genomes. Comparative genomic studies in various lineages have shown that TEs play a key role in genome diversity by expansion (Petrov et al., 2014). A genome-wide comparative sequence analysis between two rice cultivars, Nipponbare and 9311, showed that TE insertions contribute to 14% of genomic differences (Xuehui et al., 2008). Additionally, 1,664 mPing TE insertions were unraveled in 24 rice accessions (Naito et al., 2009), and 34,154 TE insertions were identified in soybeans in 31 wild and cultivated soybean varieties (Tian et al., 2012). In a recent study in rice, differentiation of TE families was identified among indica and japonica (two rice varieties) (Carpentier et al., 2019). However, this process does not always lead to genome expansion, while processes for the rapid removal of DNA from plant genomes are always occurring (Ma et al., 2004; Clémentine and Bennetzen, 2006; Dai et al., 2018). To date, a comprehensive analysis of TE repeats at the population level in maize has not been conducted. By taking advantage of next-generation sequencing data in the maize HapMap3 population and well-annotated TE sequences in reference Version4 (V4) of maize (Bukowski et al., 2015; Jiao et al., 2017), retrotransposon insertion polymorphisms (RIPs) during maize domestication and improvement were identified in this study.

TEs in maize can be classified into two super families, Class I and II, among which, the *Copia* and *Gypsy* families in Class I account for ∼90% of all TE sequences (Schnable et al., 2015; Jiao et al., 2017; Springer et al., 2018; Sun et al., 2018). This suggests that the two families play major roles in genome dynamics due to TE activity. The *Copia* and *Gypsy* families can be subdivided into more than 400 sub-families (repeat number per sub-family > = 20). The maize HapMap3 population harbors 1,218 lines, which includes teosinte, landrace, and improved categories. In this study, we applied the TRACKPOSON method in order to identify 27 representative TE families belonging to the *Copia* and *Gypsy* families in the maize HapMap3 population (Carpentier et al., 2019). Considering the large size of the maize genome, 125 lines with sequencing depths ranging from 4X to 8X were selected to identify RIPs in order to save computational resources. Here, it was demonstrated that TE insertion frequency was relatively lower in improved groups than in teosinte or landrace groups. TE copy numbers were more abundant in landrace lines than in teosinte or improved lines. Additionally, teosinte, landrace, and improved lines could be clearly distinguished by RIPs. The findings of this study serve as an important resource for the dissection of TE variation during maize domestication and improvement.

## Methods

### Database

Sequencing data were downloaded according to the SRA accessions described in a previous paper (Bukowski et al., 2015), which included 1,218 maize lines from teosinte, landrace, and improved accessions. In total, 125 maize lines were selected to analyze the TE insertions in the maize genome. The sequencing depth of 125 maize lines ranged from 4X to 8X. Similar sequencing depths eliminated bias when analyzing TE insertions. In order to construct the TE sequence dataset, the TE annotation bed file was downloaded from the MaizeGDB website. TE sequences were obtained using the BEDtools v2.25.0 software. In total, 27 TE families were retained for subsequent analyses (**Supplementary Dataset 2**).

### Identification of the TE Insertion Location

Bowtie 2 software (version 2.3.4.3) was used to align short reads to the TE reference, and was set in the very-sensitive mode (Langmead and Salzberg, 2012). SAM files were converted to a BAM file using SAMtools v1.9 software (Li et al., 2009). Paired reads, for which one paired read mapped to the TE reference, while the other was not mapped, were retained for subsequent analyses. Next, the not mapped reads were mapped to the maize reference genome using BWA-MEM software (version 0.7.17) (Li, 2013). Unique mapped reads were retained to anchor the TE insertion location. Finally, unique mapped reads were split into a 10 kb window to identify the TE insertion location. If read counts in the 10 kb window were >3, it was considered a TE insertion location.

### PCA

For PCA, the TE insertion matrix was first converted into a ped and map file using the in-house perl code. PLINK software (version 1.07) with the "–make-bed –noweb" parameter was used to calculate the genetic distances between maize lines (Purcell et al., 2007). Then, GCTA64 software (version 1.26.0) was used to analyze the PCA in two steps. The parameters of the first step were –bfile (input file) –make-grm (estimating the genetic relationships among individuals) –autosome (only considering autosome). The parameters of the second step were –grm (the format of input file) –pca 2 (output the first and second components). The first and second components were used to analyze the genetic distances between maize lines (Yang, 2013).

### Estimating TE Copy Number

For each of the 27 TE families for a given line, the TE copy number was calculated. The TE copy number was defined as the number of 10 kb windows that were covered by at least three reads.

### Calculating the TE Insertion Frequency

The TE insertion frequency was calculated as the total number of TE insertions in a 10 kb window divided by the number of accessions (like allele frequency).

### Code Availability

All the custom codes in this study are available in the **Supplementary Material Presentation 1**.

## Results

### Strategy for Identifying RIPs in 125 Maize Lines

Resequencing data for 125 lines, including 10 teosinte, 24 landrace, and 91 improved lines, were screened for RIPs following the methods described by Carpentier et al. (2019) (**Supplementary Dataset 1**). The bioinformatic pipelines included several steps: 1) Constructing a TE sequence dataset based on the maize V4 TE annotated bed file (Jiao et al., 2017); 2) Aligning the paired reads for a given accession to the TE sequence dataset; 3) Identifying the paired reads, where one paired read was mapped and the other was unmapped to the TE sequence; 4) Aligning the unmapped paired reads to the maize B73 reference genome in order to anchor the position of the TE insertion; 5) Counting the read numbers in a 10 kb window across the genome; and 6) Defining the TE insertion location if the read counts in the 10 kb window were >3.

In order to evaluate the performance of the pipeline to detect TE insertions in the maize genome, TE insertions in the Mo17 genome were tested, taking advantage of its complete sequenced genome (Sun et al., 2018). In total, 84,868 TE insertion events were discovered in Mo17, by aligning reads to the B73 reference. For each paired-end read, with one mapped to B73 (R1) and the other mapped to the TE sequence (R2), the R1 read was aligned to the Mo17 reference. Then, the sequences around the insertion position (2 kb) in Mo17 were obtained. Results revealed that 95% of the R2 reads in Mo17 could be mapped to the 2 kb reference, which indicated that the pipelines for detecting the position of the TE insertions were suitable for maize.

### Genome-Wide Identification of RIPs Belonging to 27 Families in 125 Maize Lines

The maize HapMap3 contained 1,218 lines of teosinte, landrace, and improved groups. The sequencing depth was 4.51X on average. In order to eliminate bias caused by sequencing depth, 125 lines were analyzed, which included 10 teosinte, 24 landrace, and 91 improved lines (**Supplementary Dataset 1**). The sequencing depth of the 125 lines ranged from 4X to 8X (**Supplementary Dataset 1**; **Figure S1**). The maize B73 genome was annotated with more than 400 retrotransposon families (repeat number per sub-family > = 20). Among them, 27 families were considered in subsequent analyses (**Supplementary Dataset 2**). The copy number of the 27 families ranged from 26 to 16,072, and the total size per family ranged from 269,895 to 250,033,953 bp (overlapped sequence), which represented the complete range of the retrotransposon sequence in maize.

**Figure 1:**
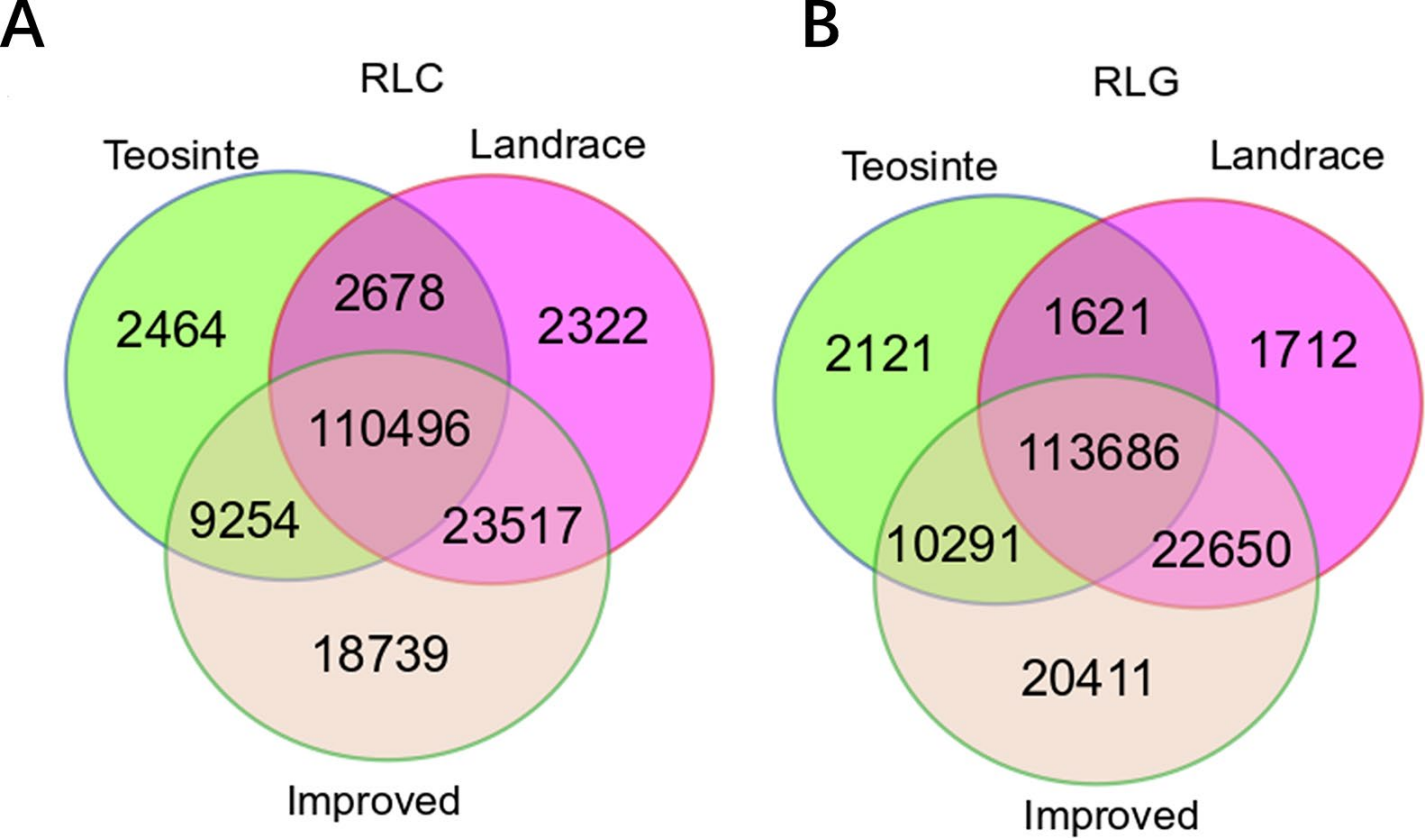
Copy number of retrotransposon elements identified in 125 maize lines. A Venn diagram illustrates the overlap of the TE insertion in the teosinte, landrace, and improved groups, respectively. **(A)** *Copia* family.**(B)** *Gypsy* family.

The positions of RIPs in teosinte, landrace, and improved genomes were mapped. In total, 124,892, 139,013, and 162,006 RIPs were identified in the *Copia* family, and 127,719, 139,669, and 167,038 RIPs in the *Gypsy* family in teosinte, landrace, and improved groups, respectively (**Figures 1A, B**). In the three groups, 110,496 (65.2%) RIPs in the *Copia* family and 113,686 (65.9%) in the *Gypsy* family were detected. Moreover, 2,464 (1.43%), 2,322 (1.37%), and 18,739 (10.84%) unique RIPs in the *Copia* family and 2,121 (1.23%), 1,712 (0.99%), and 20,411 (11.83%) unique RIPs in the *Gypsy* family were detected in teosinte, landrace, and improved groups, respectively. The unique RIPs were more abundant in the improved groups than the teosinte or landrace groups. In the analyses, 75% of detected lines were improved lines, which might result in more abundant RIPs in the improved groups.

**Figure 2:**
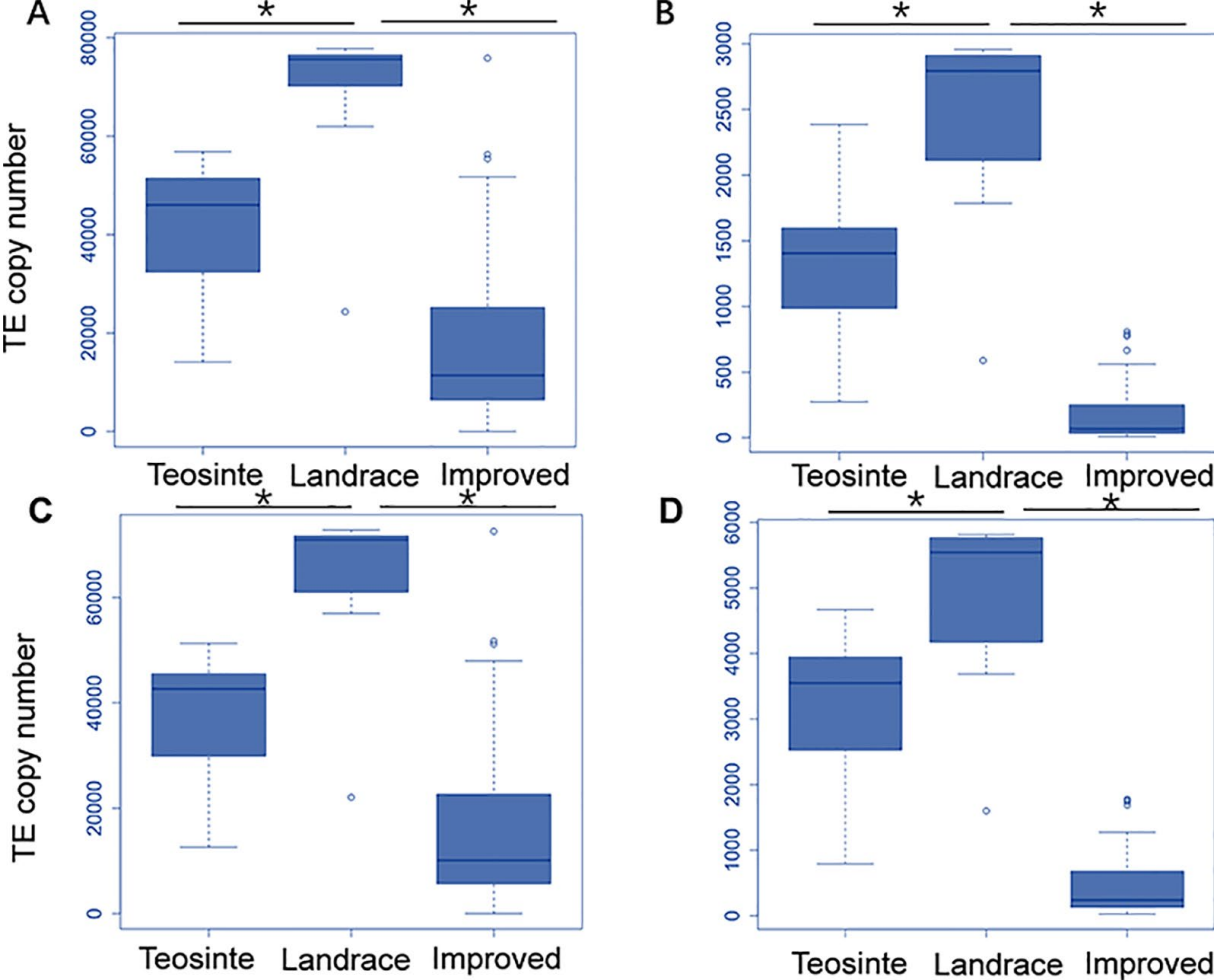
Distribution of the TE copy number in the teosinte, landrace and improved groups. We plotted the distribution of the TE copy number for four families. The y-axis represents the TE copy number for all accessions in each group. **(A)** *ji family*. **(B)***debeh family*. **(C)** *cinful-zeon family*. **(D)** *CRM2 family.* * represented p value < = 0.05.

Next, the averaged TE copy number was analyzed for a given group. Results revealed that all 27 TE families showed variable copy numbers in each of the three groups (**Table 1**). Copy numbers ranged from 1,223 to 40,481, 2,397 to 67,907, and 164 to 17,162 in the teosinte, landrace, and improved groups, respectively (**Table 1**). Interestingly, the highest copy number of TE insertions for both the *Gypsy* and *Copia* families was detected in landrace accessions, followed by teosinte and improved accessions (**Figure 2**). These results suggest that the 27 families of retrotransposons were amplified during maize domestication and contracted during maize improvement.

**Table 1:** TE insertion summary of the 27 families.

Family	Superfamily	Mean insertion in teosinte groups	Mean insertion in landrace groups	Mean insertion in improved groups
**ji**	*Copia*	40481	67907	17162
**opie**	*Copia*	33401	57978	14100
**ruda**	*Copia*	19117	31876	4692
**giepum**	*Copia*	15259	26783	4258
**wiwa**	*Copia*	6746	11720	1363
**ebel**	*Copia*	7720	14345	1619
**gudyeg**	*Copia*	9919	17833	2646
**machiavelli**	*Copia*	6024	11654	1814
**raider**	*Copia*	2240	3719	356
**debeh**	*Copia*	1315	2397	164
**japov**	*Copia*	1223	2583	165
**cinful-zeon**	*Gypsy*	36626	62768	15515
**huck**	*Gypsy*	34127	58101	14642
**xilon-diguus**	*Gypsy*	26686	45344	8911
**flip**	*Gypsy*	28796	49505	10303
**grande**	*Gypsy*	28108	48938	10405
**doke**	*Gypsy*	21497	38410	7977
**gyma**	*Gypsy*	26213	44109	7278
**milt**	*Gypsy*	21227	37335	7044
**dagaf**	*Gypsy*	17152	30175	5018
**puck**	*Gypsy*	19598	34674	6373
**uwum**	*Gypsy*	9568	15810	1902
**CRM1**	*Gypsy*	8644	15300	1973
**tekay**	*Gypsy*	15403	25904	3559
**CRM4**	*Gypsy*	11905	19853	2572
**CRM2**	*Gypsy*	3144	4797	432
**guhis**	*Gypsy*	8255	13990	1893

### The Significant Difference of TE Insertion Frequency Among Teosinte, Landrace, and the Improved Groups

**Figure 3:**
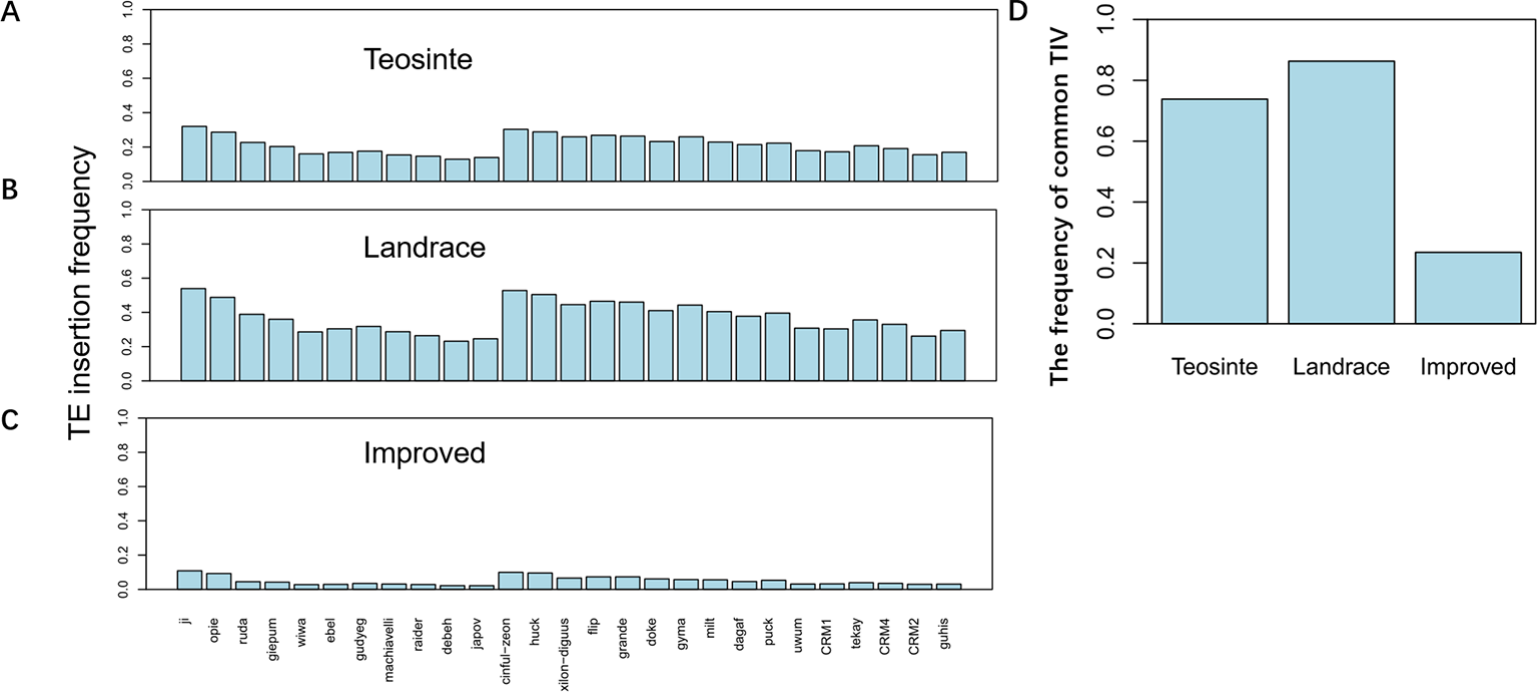
TE insertion frequency for 27 families. The average TE insertion frequency for the **(A)** teosinte, **(B)** landrace, and **(C)** improved groups. **(D)** The frequency of common RIPs in the three groups. The y-axis represents the percentage of RIPs (insertion frequency > 0.2).

TE insertion frequency (TE insertions/10kb window/number of accessions) was calculated for 27 families in each of the three groups. Results revealed that TE insertion frequency, on average, was about 0.3, 0.5, and 0.1 in the teosinte, landrace, and improved groups, respectively (**Figures 3A–C**). The highest TE insertion frequency occurred in the landrace groups, followed by the teosinte and improved groups. Furthermore, the TE insertion frequency of 27 families did not exhibit a similar level and some families exhibited very low frequency. For example, the insertion frequency of the *jopoa* family was about 0.02 in the improved groups (**Figure 3C**). The frequency of common RIPs was also analyzed (the insertion frequency was above 20% for detected lines). Results revealed that the common RIPs occupied about 20%, 80%, and 10% of the teosinte, landrace, and improved groups, respectively (**Figure 3D**). These results demonstrated that TE insertion frequency was increased and reduced during maize domestication and improvement, respectively.

### Teosinte, Landrace, and Improved Groups Can Be Clearly Distinguished by RIPs

**Figure 4:**
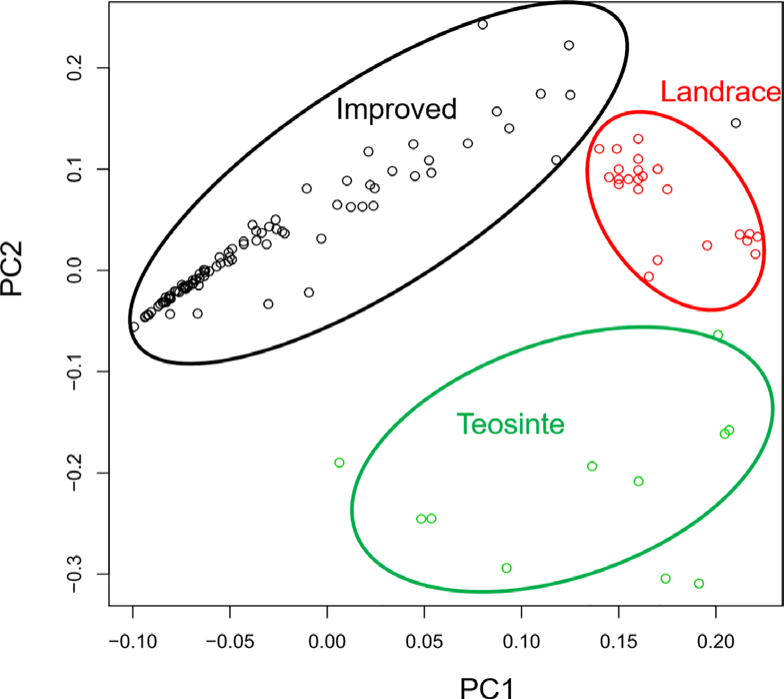
Principal component analysis for 125 maize lines.

In a previous study, the teosinte, landrace, and improved groups could be distinguished by SNPs (Hufford et al., 2012). Thus, this study aimed to determine whether the three groups could also be distinguished by RIPs. RIP maps were constructed for all detected lines, and the genetic distances of these lines were calculated by PCA. The distribution of the 125 maize lines was plotted by PC1 and PC2 (**Figure 4**). Interestingly, the three populations were distinguishable by RIPs (**Figure 4**). The lines in the landrace group were clustered tightly, while the lines in the teosinte and improved groups were more dispersed (**Figure 4**).

### Insertion Distribution of 27 TE Families Across the Genome

**Figure 5:**
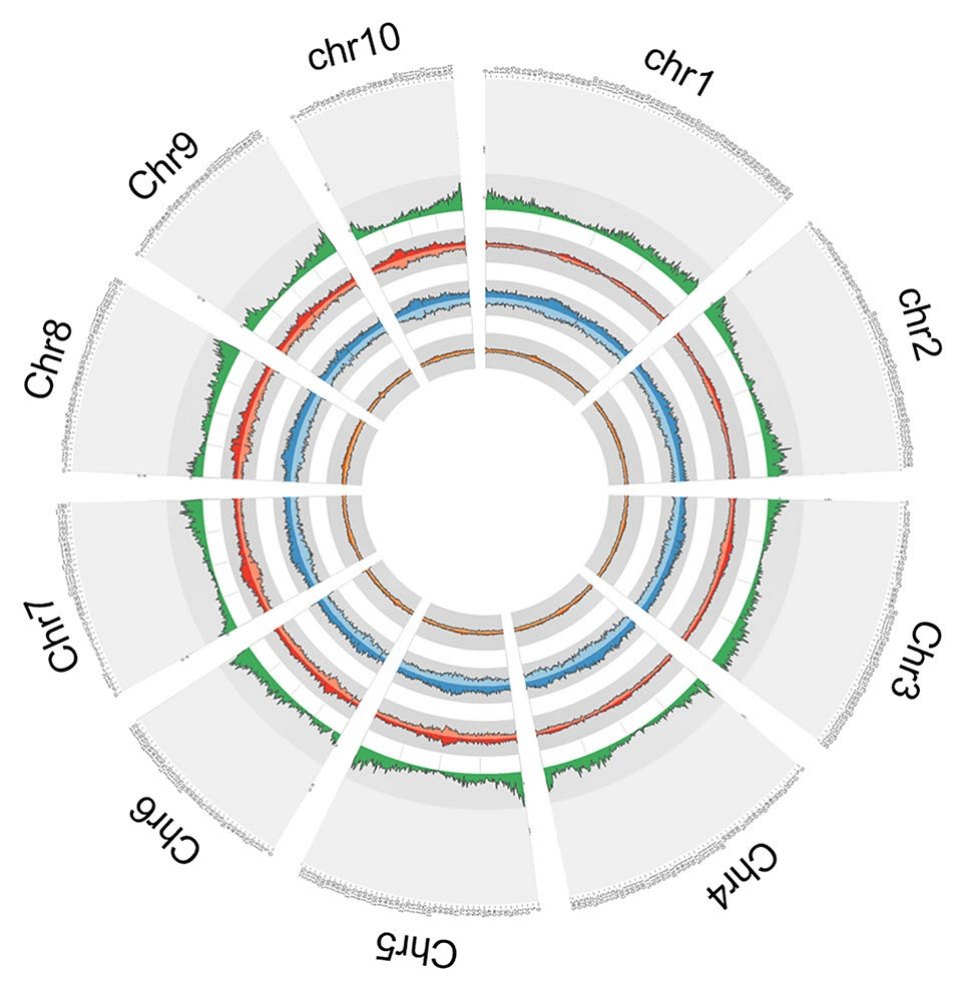
Circular representation. From outside to inside: the first circle represents the gene density; the second circle represents the *Copia* (dark red) and *Gypsy* (light red) in the teosinte groups; the third circle represents the *Copia* (dark blue) and *Gypsy* (light blue) in the landrace groups; the forth circle represents the *Copia* (dark green) and *Gypsy* (light green) in the improved groups.

**Figure 6:**
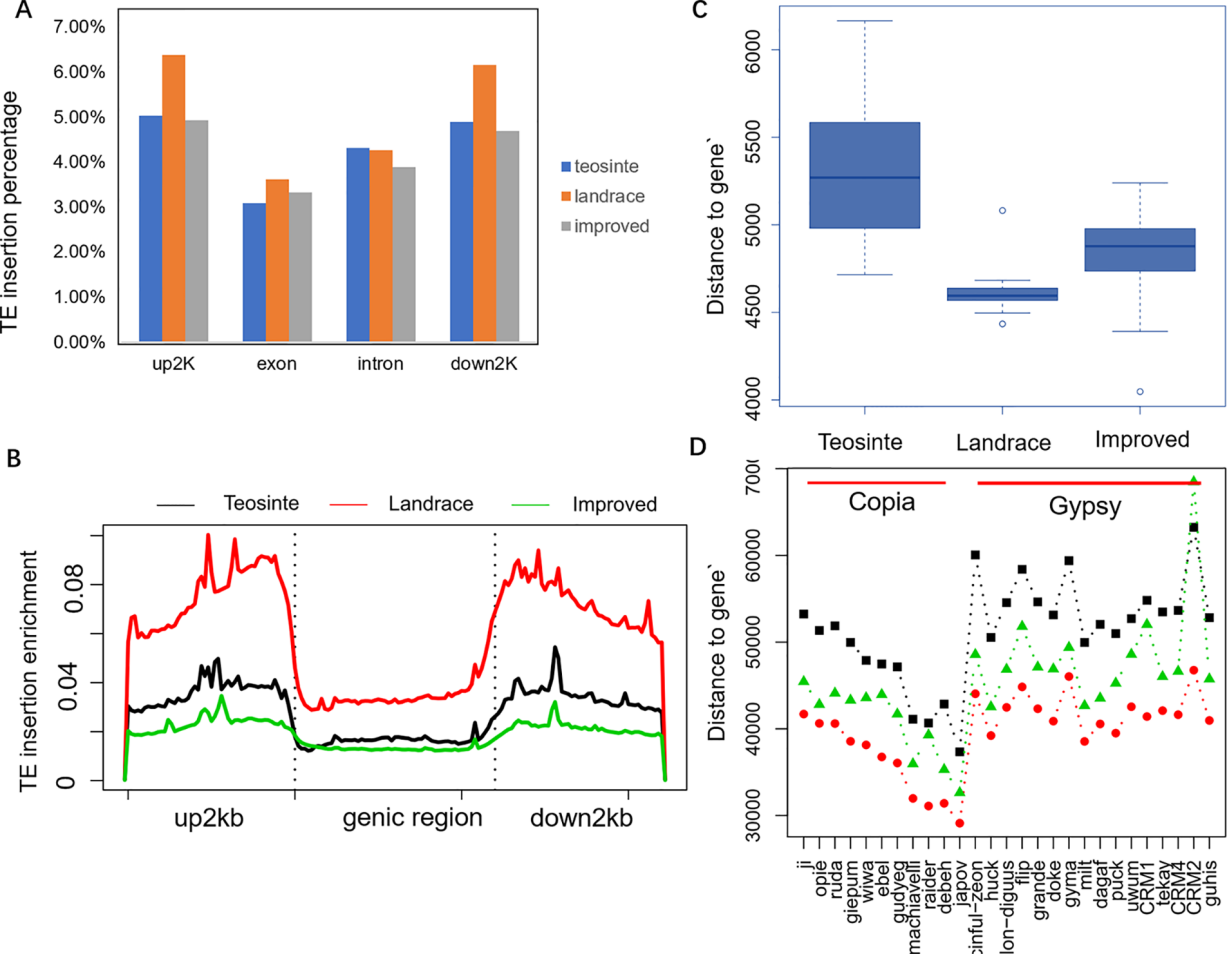
TE insertion distribution across genome features. **(A)** The TE insertion percentage in upstream 2 kb (up2kb), downstream 2 kb (down2kb), and the exon and intron region of the gene in the teosinte, landrace, and improved groups, respectively. **(B)** TE insertion profiling around the genic region. The y-axis represents the normalized TE reads. **(C)** The distance of the TE insertion to genes. **(D)** The distance of the TE position to genes for each of the 27 families.

In order to explore the insertion distribution of 27 TE families, the TE insertion number and frequency in a 1 Mb window were calculated, and their distribution patterns with gene density along chromosomes was compared. The mapping data showed that no region across the genome lacked TE insertion (**Figure 5**; **Figure S2**), suggesting that TE insertions contributed to genome-wide diversity along the chromosome. For the *Copia* family, the correlation values between TE insertion number and gene density were -0.305, -0.362, and -0.127 for teosinte, landrace and teosinte, respectively. For the *Gypsy* family, the correlation values between TE insertion number and gene density were -0.334, -0.428, and -0.300 for teosinte, landrace, and teosinte, respectively (**Figure S3**). These results demonstrate that the TE insertion number for the *Copia* and *Gypsy* families was negatively correlated with gene density and the landrace groups showed the highest negative correlation, followed by the teosinte and improved groups. Clearly, the highest TE insertion density was located around the centromeric regions (**Figure 5**; **Figure S2**). However, in the improved groups, TE insertions were less abundant in the centromeric regions. In order to further analyze the distribution of TE insertions in detail, the TE distribution in each exon, intron, and 2 kb up- and downstream of genes, and intergenic region was calculated (**Figure 6A**). Results revealed that TE insertion in the intergenic region occupied 82.6%, 79.5%, and 83.1% of the teosinte, landrace, and improved groups, respectively. For the landrace groups, TEs were less abundant in the intergenic region. TE insertions in the 2 kb up- and downstream of genes accounted for 9.93%, 12.54%, and 9.61% of the teosinte, landrace, and improved groups, respectively. TEs were more abundant in the landrace groups in these regions (**Figure 6A**).

The profile of TE insertions around the genic region was detected in detail (**Figure 6B**). Results revealed that TE insertions were preferably located upstream and downstream of the genic region. Landrace accessions showed more enrichment upstream and downstream of the genic region compared to the teosinte and improved accessions. TE insertions were more abundant in the upstream and downstream regions but less abundant in the intergenic region of the landrace groups, which may indicate that the domestication process was related to a regulatory region variation.

Furthermore, the distances of TE insertions to the transcriptional start site (TSS) were analyzed. Overall, the average distances to TSS were 5,200, 4,500, and 4,800 bp for the teosinte, landrace, and improved groups, respectively, which demonstrates that TE insertions in the landrace group were closer to TSS (*P* < 0.01, T-test) (**Figure 6C**). The distances of the 27 families were further analyzed in detail. Overall, in the landrace groups, TEs were closer to the genes compared to the other groups in almost all 27 families, except the *CRM2* family (**Figure 6D**). TEs in the *Copia* family were closer to genes than the *Gypsy* family. These results indicate that the distances of TE insertions to TSS varied among families.

It was obvious that TEs were more abundant upstream of the genic region in the landrace groups compared to the teosinte groups (**Figure 6C**). One-thousand-nine-hundred-and-twenty genes were identified, which exhibited more TE enrichment (Reads depth: Landrace/teosinte> = 5) upstream of the genic regions. Through gene ontology enrichment analysis, these 1920 genes were enriched for basal cellular functions, such as protein modification processes, chromatin assembly, nucleosome assembly, and ATP binding. These results suggest that basal cellular pathways are involved in the maize domestication period

## Discussion

### TE Loss During Maize Improvement

TEs can cause new mutations through random insertions in the genome, which contributes to larger genome sizes (Brunner et al., 2005). In this study, TEs were found to be relatively more abundant in the landrace than in the improved accessions, indicating TE loss during the maize improvement process (**Table 1**; **Figure 2**). In previous studies, the LTR family in rice was analyzed to learn about the variation of their structures. It was found that more than 75% of LTRs were not intact (Ma et al., 2004). Thus, it was concluded that unequal homologous recombination and illegitimate recombination lead to LTR depletion (Ma et al., 2004). Therefore, it is clear that during the maize improvement process, the maize genome is resized through TE expansion and removal.

### Relatively Lower TE Insertion Frequency in Improved Maize Than in Teosinte or Landrace Accessions

The insertion frequency of the 27 retrotransposon families was relatively lower in improved accessions than in teosinte or landrace accessions (**Figure 3**). Lower RIP frequencies have also been observed in *Arabidopsis thaliana* (Quadrana et al., 2016), as well as rice (Carpentier et al., 2019). Most RIPs were unique or shared by two accessions in the improved lines, indicating that genome diversification driven by TE was ongoing during maize breeding.

## Conclusion

TEs are a key genetic component discovered in the maize genome that can cause mutation by disrupting the expression level of gene or chromosome rearrangement. However, to date, a detailed analysis of different retrotransposon element families in maize has not been conducted. In this study, 27 families were analyzed during maize domestication and improvement. Results revealed that the TE copy number was more abundant in landrace accessions than in teosinte or improved accessions for 27 TE families. Additionally, TEs were more abundant in the centromeres along the chromosomes in landrace groups than in improved groups. TEs were more enriched in the promoter region in landrace accessions compared with teosinte or improved accessions. These results demonstrate that TEs were amplified and contracted during maize domestication and improvement, respectively.

## Data Availability Statement

Publicly available datasets were analyzed in this study. This data can be found here: PRJNA389800.

## Author Contributions

YQ and XZ designed the study and analyzed the data. XZ wrote the paper.

## Funding

This work was supported by the China Agricultural Research System (CARS201707); the Guangdong Academy of Sciences Special Funds for Building Top-ranking Research Institutions in China (2019GDASYL-0104013).

## Conflict of Interest

The authors declare that the research was conducted in the absence of any commercial or financial relationships that could be construed as a potential conflict of interest.
