# Supplementary material for: The Landscape of Copia and Gypsy Retrotransposon During Maize Domestication and Improvement
Source: Front Plant Sci. 2019 Dec 18;10:1533. doi: 10.3389/fpls.2019.01533 (PMC6930232; doi:10.3389/fpls.2019.01533)
Supplement: Supplementary file 1 [file DataSheet_1.pdf]

Supplementary Material

Supplementary Figure 1

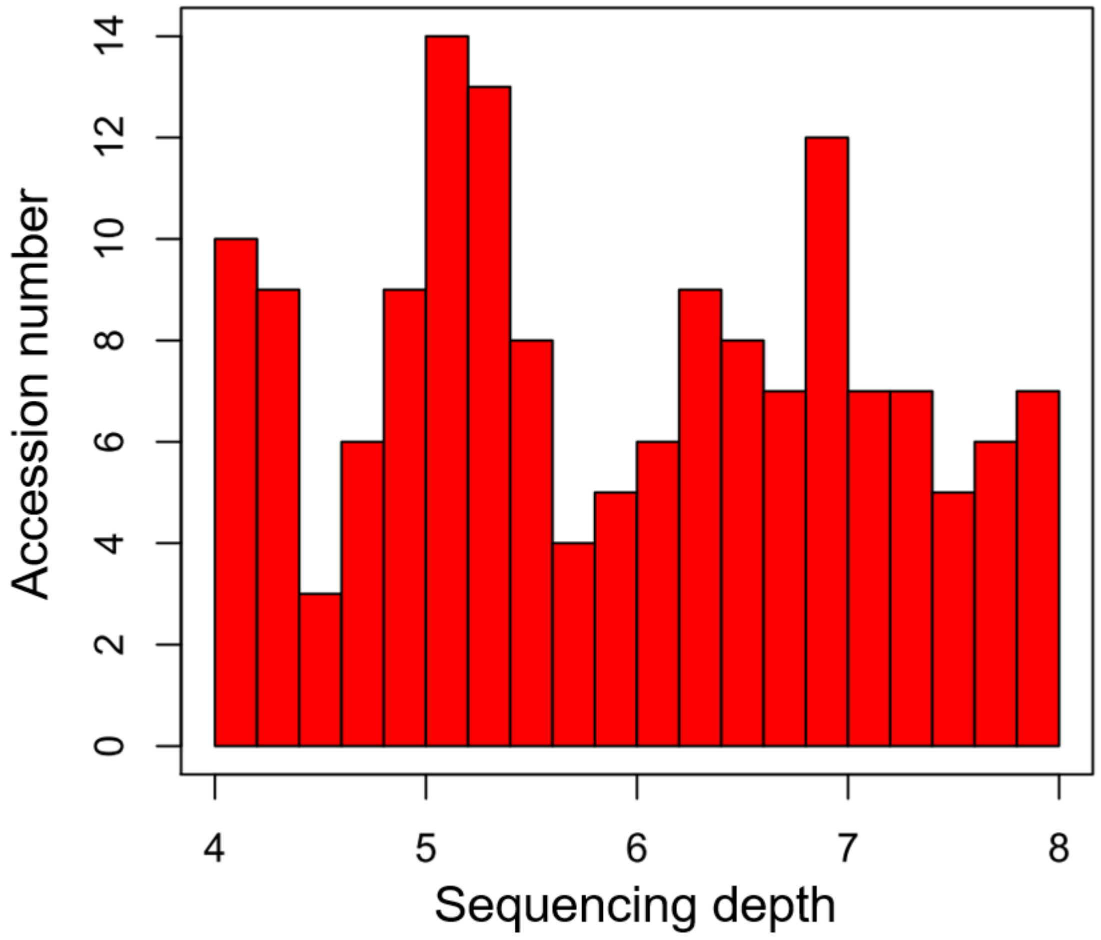

Sequencing depth of 125 maize lines used to identity RIPs.

Supplementary Figure 2

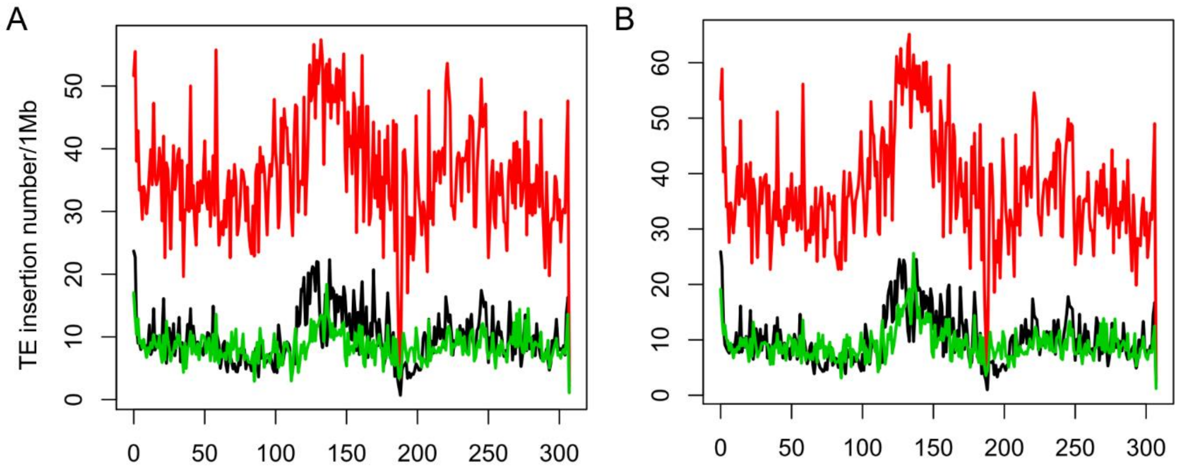

The TE insertion number across chromosome 1. The distribution of the *Copia* (A) and *Gypsy*(B) superfamily on chromosome 1, Black line: teosinte groups; Red line: landrace groups; Green line: improved groups.

Supplementary Figure 3

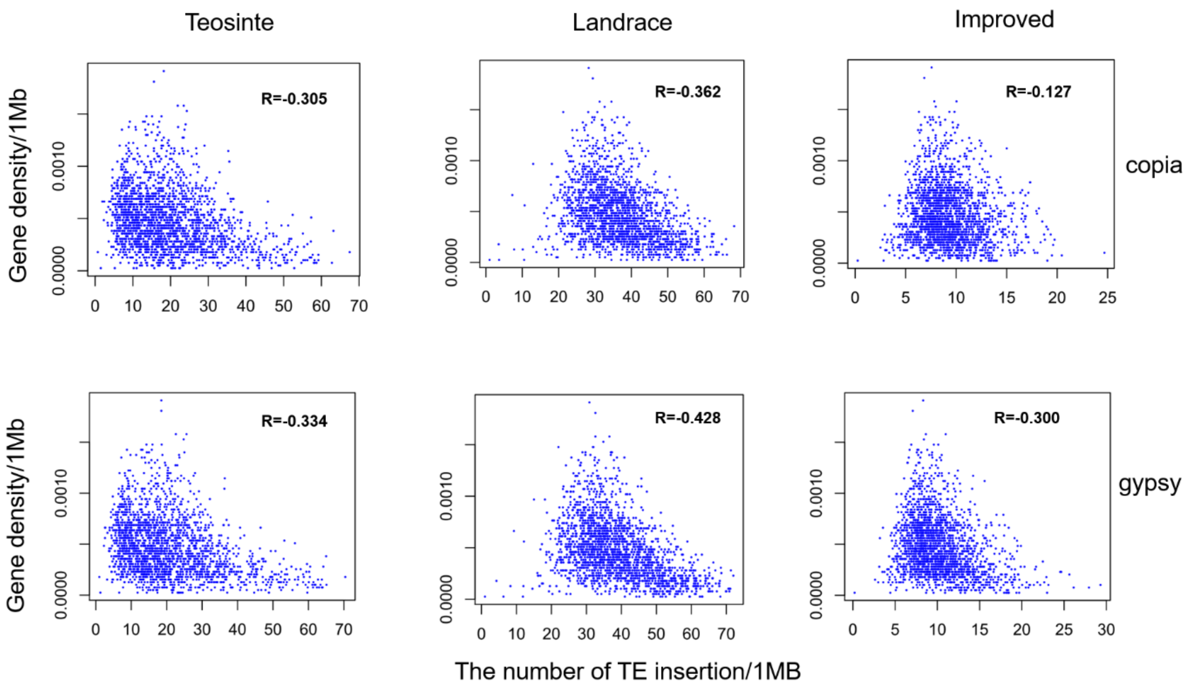

Correlation between gene density and the TE insertion.

Supplementary Dataset 1

Information on the 125 lines used in this study to identify RIPs.

species	lines	SRS accession	sequencing depth
improved	282set_A6	SRS2266928	4.825166135
improved	282set_A659	SRS2267002	5.531993555
improved	282set_A680	SRS2267000	7.374417775
improved	282set_A682	SRS2266934	7.328377615
improved	282set_Ab28A	SRS2266932	4.604901953
improved	282set_B10	SRS2266865	4.035761435
improved	282set_B103	SRS2266931	7.159068598
improved	282set_B105	SRS2266930	4.57087272
improved	282set_B109	SRS2266864	6.790684735
improved	282set_B14A	SRS2266862	5.970521445
improved	282set_B37	SRS2266873	6.989110406
improved	282set_B46	SRS2267040	6.194257091
improved	282set_B52	SRS2267039	4.073240864
improved	282set_B64	SRS2267041	6.436989363
improved	282set_B77	SRS2266967	4.769410338
improved	282set_C103	SRS2266889	7.312032348
improved	282set_C49A	SRS2266893	5.052123627
improved	282set_CH9	SRS2266898	7.145983453
improved	282set_CI21E	SRS2266823	5.449912022
improved	282set_CI28AGoodman-Buckler	SRS2266824	4.293697713
improved	282set_CI31A	SRS2266825	5.031186531
improved	282set_CI3A	SRS2266826	6.430036742
improved	282set_CI64	SRS2266827	6.560718364
improved	282set_CI66	SRS2266829	7.729917394
improved	282set_Ci7Goodman-Buckler	SRS2266831	4.730260512
improved	282set_Ci91BGoodman-Buckler	SRS2266900	6.180142362
improved	282set_CM105	SRS2266820	7.419587205
improved	282set_CM37	SRS2266822	5.195093059
improved	282set_CM7	SRS2267016	6.984082527
improved	282set_CML10	SRS2267013	4.733382611
improved	282set_CML108	SRS2267014	7.880929573
improved	282set_CML11	SRS2267012	4.345452655
improved	282set_CML14	SRS2267011	6.937967335
improved	282set_CML154Q	SRS2266945	4.337423018
improved	282set_CML218	SRS2266948	5.220704055
improved	282set_CML220	SRS2266949	5.982245925
improved	282set_CML228	SRS2267025	6.551451835
improved	282set_CML247	SRS2267024	6.529724391
improved	282set_CML254	SRS2267022	6.40621178
improved	282set_CML261	SRS2266793	4.515595663
improved	282set_CML264	SRS2266792	5.918212574
improved	282set_CML277	SRS2266790	6.837894282
improved	282set_CML281	SRS2266789	7.537614043

improved	282set_CML287	SRS2266794	4.821421514
improved	282set_CML314	SRS2266881	7.305256432
improved	282set_CML321	SRS2266878	4.066393844
improved	282set_CML322	SRS2266879	5.517777445
improved	282set_CML323	SRS2266882	6.305891743
improved	282set_CML328	SRS2266978	7.613697753
improved	282set_CML332	SRS2266976	4.269720809
improved	282set_CML333	SRS2266977	7.183311422
improved	282set_CML38	SRS2266778	7.038772665
improved	282set_CML45	SRS2266777	7.070594864
improved	282set_CML5	SRS2266775	6.059494676
improved	282set_CML52	SRS2266776	7.820233346
improved	282set_CML61	SRS2266780	6.216278239
improved	282set_CML69	SRS2266779	6.973140281
improved	282set_CML92	SRS2266834	5.266878136
improved	282set_CO106	SRS2266842	7.878937091
improved	282set_CO125	SRS2266837	7.723939364
improved	282set_DE1	SRS2266915	7.890993679
improved	282set_DE-2	SRS2266902	6.86752786
improved	282set_DE811	SRS2266897	5.495608445
improved	282set_EP1	SRS2266874	4.092549709
improved	282set_F2834T	SRS2266876	5.351154292
improved	282set_F7	SRS2266783	6.248616062
improved	282set_GA209	SRS2266787	6.856958424
improved	282set_GT112	SRS2266939	4.151244777
improved	282set_H105W	SRS2266940	6.628328773
improved	282set_H49	SRS2266938	7.363028438
improved	282set_H84	SRS2266941	6.225030662
improved	282set_Hi27Goodman-Buckler	SRS2267038	5.69926668
improved	282set_i1677a	SRS2266999	6.898684763
improved	282set_IA2132Goodman-Buckler	SRS2266959	4.039627628
improved	282set_K148	SRS2266850	4.158173752
improved	282set_K4	SRS2266848	6.750465682
improved	282set_Ki11	SRS2266785	6.661043279
improved	282set_Ki14	SRS2266788	4.626037009
improved	282set_Ki2021	SRS2266984	4.104371178
improved	282set_Ki21	SRS2266985	7.788179177
improved	282set_Ki44	SRS2266981	5.15644203
improved	282set_Ky21	SRS2266986	6.711654659
improved	282set_KY226	SRS2266786	4.230287955
improved	282set_M14	SRS2266912	6.794588077
improved	282set_M162W	SRS2266911	7.113702624
improved	282set_M37W	SRS2266905	5.979213767
improved	282set_Mo17	SRS2266845	6.937148299
improved	282set_Mo18W	SRS2266867	5.070659655
improved	282set_MO1W	SRS2266903	6.350897876
improved	282set_Mo24W	SRS2266866	4.885832261
improved	282set_Mo46	SRS2267032	5.682567766
landrace	BKN009	SRS302389	5.215737364

landrace	BKN010	SRS302390	5.087290545
landrace	BKN011	SRS302391	5.323762273
landrace	BKN014	SRS302392	5.239093182
landrace	BKN015	SRS302393	5.384026545
landrace	BKN016	SRS302394	5.054460364
landrace	BKN017	SRS302395	5.064310364
landrace	BKN018	SRS302396	4.922012636
landrace	BKN019	SRS302397	4.822171455
landrace	BKN020	SRS302398	5.195373545
landrace	BKN022	SRS302399	5.225883909
landrace	BKN023	SRS302400	5.164316273
landrace	BKN025	SRS302401	5.141250909
landrace	BKN026	SRS302402	5.162910636
landrace	BKN027	SRS302403	5.266424909
landrace	BKN029	SRS302404	5.273097636
landrace	BKN030	SRS302405	5.220271
landrace	BKN031	SRS302406	5.100959818
landrace	BKN032	SRS302407	4.779915636
landrace	BKN033	SRS302408	4.998452545
landrace	BKN034	SRS302409	4.853961
landrace	BKN035	SRS302410	4.928927273
landrace	ZEAxppRCWDIAAPEI-3	SRS2455568	5.587839564
landrace	ZEAxppRCXDIAAPEI-4	SRS2455567	5.388894545
teosinte	TIL01-JD	SRS2466045	6.387713446
teosinte	TIL03	SRS2466048	6.684110342
teosinte	TIL04-TIP454	SRS302465	4.275851516
teosinte	TIL05	SRS302466	5.379134073
teosinte	TIL09	SRS302471	4.264493415
teosinte	TIL10	SRS2466049	7.966526727
teosinte	TIL11	SRS2466044	6.087978675
teosinte	TIL14-TIP498	SRS2466050	6.215441531
teosinte	TIL15	SRS302476	5.603795538
teosinte	TIL25-TIP489	SRS2466037	6.254726307

Supplementary Dataset 2

Copy and sequence size of the 27 TE families are analyzed in this study.

Code	Family name	family	Copy number	Genome size
RLC00002	ji	Copia	15,175	234,912,133
RLC00004	opie	Copia	12,011	158,065,343
RLC00013	ruda	Copia	1,017	10,236,941
RLC00019	giepum	Copia	567	9,989,185
RLC00023	wiwa	Copia	504	4,511,656
RLC00032	ebel	Copia	329	2,613,741
RLC00034	gudyeg	Copia	299	3,480,214
RLC00046	machiavelli	Copia	175	1,898,054
RLC00052	raider	Copia	121	839,105
RLC00143	debeh	Copia	27	269,895
RLC00148	japov	Copia	26	306,463
RLG00001	cinful-zeon	Gypsy	16,072	201,429,757
RLG00003	huck	Gypsy	12,861	250,033,953
RLG00005	xilon-diguus	Gypsy	4,387	61,285,846
RLG00006	flip	Gypsy	4,533	79,126,276
RLG00007	grande	Gypsy	3,256	68,481,539
RLG00008	doke	Gypsy	2,994	46,930,607
RLG00009	gyma	Gypsy	1,958	32,196,812
RLG00011	milt	Gypsy	1,296	18,372,537
RLG00017	dagaf	Gypsy	672	10,673,837
RLG00018	puck	Gypsy	587	11,428,694
RLG00020	uwum	Gypsy	600	5,137,928
RLG00024	CRM1	Gypsy	475	4,614,708
RLG00028	tekay	Gypsy	416	7,052,848
RLG00035	CRM4	Gypsy	295	3,584,328
RLG00036	CRM2	Gypsy	234	2,093,115
RLG00038	guhis	Gypsy	229	2,783,250
